# Toxic substances in hearts of children with cardiovascular malformations

**DOI:** 10.1186/1749-8090-8-S1-O139

**Published:** 2013-09-11

**Authors:** O Koval, I Mokryk, N Nagorna, G Dubova, O Bordyugova, A Novack, O Pshenychnaya, R Klymanskiy, O Kartashova, V Konov

**Affiliations:** 1 Pediatric Subdepartment of Internship and Postgraduate Education Faculty of Donetsk National Medical University n.a. M. Gorkiy, Donetsk, Ukraine; 2 Department of Cardiosurgery, Cardiology and Rehabilitation for Children, Government Institution “Institute of Urgent and Recovery Surgery named after V. K. Gusak National Academy of Medical Science of Ukraine”, Donetsk, Ukraine

## Background

According to experimental data, numerous substances (including toxic, potentially toxic, conditionally essential chemical elements) have ability to pass through the placental barrier and disrupt cardiogenesis. The aim of this research was to study the level of toxic substances content in heart tissue samples of children with CHD.

## Methods

29 children (17 boys and 12 girls), aged from 14 days to 17 years old with different CHD underwent content analysis of following toxic substances: Al, Cd, Pb, Hg, Be, Ba, Tl, Bi, As, Ni, Sb , Sn, Sr, Ti, W, Zr, Ag, Li, B, Co, Si, V in intraoperative (23) and autopsy (6) biopsy tissue samples of heart (n=37) and great vessels (n=18) by atomic emission and absorption spectrometry methods in inductively coupled plasma. Study received an approval of ethic committee of the clinic.

## Results

Excess of permissible level of six toxic elements was revealed in myocardium, endocardium, pericardium and aortic wall tissue samples of 79.3% (n=23) of patients. Excess of permissible toxic barium content had 72.4% of patients; conditionally essential lithium content – 20.7%; toxic aluminum content – 17.2%; potentially toxic nickel content – 6.8%; strontium and arsenic content – 3.5%. Excess rate of these elements ranged from two to ten or more times and depended on biopsy points. Namely, in aortic coarctation, valve atresia, septal defect areas their level was higher than in normal areas of heart or great vessel.

## Conclusions

Our results indicate that, children with CHD have a special elevated set of specific toxic substances at the site of malformation. The above mentioned suggests a possible role of barium, aluminum, lithium, nickel, strontium, arsenic in formation of cardiovascular malformations, that needs further evaluation.

